# The optical measurement of large cluster tracks in a gas jet

**DOI:** 10.1038/srep32391

**Published:** 2016-08-26

**Authors:** Zhiyuan Chen, Dong Liu, Jifeng Han, Lixin Bai

**Affiliations:** 1 Key Laboratory of Radiation Physics and Technology of the Ministry of Education, Institute of Nuclear Science and Technology, Sichuan University, Chengdu 610064, China

## Abstract

We propose an optical method based on Rayleigh scattering for the direct measurement of cluster tracks produced by a high-pressure gas jet. The tracks of the argon and methane clusters are acquired by a high-speed camera. It is found that the cluster sizes of these tracks are within the range of 7E + 03~1E + 07 for argon and 2E + 06~4E + 08 for methane. Most argon tracks are continuous and their intensity changes gradually, while the majority of the methane tracks are separated into discrete fractions and their intensity alters periodically along the flight path, which may indicate the methane clusters are more unstable and easily to break up. Special methane clusters which may fly at an axial velocity of less than 2.5m/s are also found. This method is very sensitive to large gas cluster and has broad application prospects in cluster physics.

Clusters are made of atoms or molecules by physical or chemical bonding forces. The interaction of an intense short-pulsed laser with Nano-sized clusters has been a popular topic of study^1^ for its potential application in neutron sources^2^, X-ray sources^3^, fast electrons and ions^4^, *etc*. The supersonic gas jet is produced by opening one nozzle to inject the high pressured gas into the vacuum, the temperature of the gas decreases greatly during the free expansion process and the clusters are formed by Van der Waals forces. The clusters are produced by gas jet technique and its size and density distribution are required to analyze the laser-cluster interaction dynamics or calculate the yield rates. The cluster size is distributed over a broad range from two (dimer) up to many thousands, which can be well approximated by the log-normal function^5^,^6^. Typically the average cluster size and density^7^ are used to represent the distribution and normally the average parameters are investigated by the Rayleigh scattering method combining other techniques such as mass spectroscopy^8^, time of flight mass spectrometer^9^, and electron diffraction^10^, *etc*. Although large clusters are preferred for ensuring higher efficiency and yield rate, there are few studies of large clusters^6^,^11^ which are rare and hard to detect.

In this study, an optical method based on Rayleigh scattering is presented to measure the cluster tracks in the gas jet and large argon and methane clusters were discovered. This imaging method can be used to capture the images of cluster tracks and study the cluster interactions with materials, thereby demonstrating broad prospects for application in cluster physics.

Traditionally for the axisymmetric gas expansion, the average cluster size N_c_ (the average number of atoms per cluster) has been characterized by the semi-empirical parameter introduced by Hagena^12^,^13^,^14^,^15^, and it is revised in ref. 18 for very big clusters, which is expressed as Formula (1).


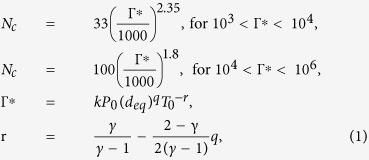


Г* is a semi-empirical parameter that takes into account all the factors^12^,^16^ affecting the average cluster size, such as the gas property (k), the nozzle geometry (deq) and the gas parameters (P_0_, T_0_). k is a gas-dependent constant (k~1650 for Ar and 2360 for CH_4_), the equivalent nozzle diameter d_eq_ is assumed to be 0.74d/tanα for monatomic clusters and 0.87d/tanα for diatomic clusters where d is the throat diameter of the nozzle in μm and α is the half opening angle of the nozzle^17^. T_0_ is the gas stagnation temperature in Kelvin, and P_0_ is the stagnation pressure in mbar. The parameter q has commonly been fixed at 0.85 in the scaling law of Hagena^13^,^17^. The exponent r is the function of q and γ, where γ is the specific heat ratio of the gas which is 5/3 for monatomic gas and 7/5 for diatomic gas respectively^18^,^19^.

However, it must be declared that the Hagena scaling law is not suitable for methane cluster. According to the results from Akiyoshi Murakami *et al*.^20^, the relation between N_c_ and Г* for methane is described in formula (2).


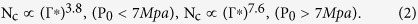


The exponent value used to calculate Nc in formula (2) is quite different from that of Hagena, and it is found that the value increases from 3.8 to 7.6 for higher stagnation pressures which is contradictory to Hagena’s formula whose exponent value decreases from 2.35 to 1.8 for higher stagnation pressure P_0._ Song Li *et al*.^21^ calculated the average methane cluster size by measuring the Coulomb explosion method, and it is reported that the average methane cluster size N_c_ = 1235 when Г* = 520 (d_eq_ = 3.85mm, P_0_ = 30bar, T_0_ = 296K, Γ^*^ ∝ P_0_(T)^−3.3^) and N_c_ = 6230 when Г* = 1104(P_0_ = 30bar, T_0_ = 240 K), and it can be calculated that the exponent value is about 2.15 for these data. It is evident that the above relations between N_c_ and Г* for methane are not accordant and it is different from the relation of N_c_ ∝ (Γ^*^)^5.78^ which is measured by our experiment and is described in the following “Results and discussion” section. So it can be concluded that the Hagena scaling law is unsuitable for methane cluster, and the exact formula is expecting for further research. In addition, the results of methane cluster size given here are subject to the date from our experiment.

The Rayleigh scattering method has been widely applied for cluster-size evaluations^22^ because it is nondestructive and relatively easy to operate. The scattering signal (S) is proportional to the cluster number density (n_c_) and the square of the average cluster size (N_c_) if the clusters are supposed to have a spherical structure^23^. The cluster number density (n_c_) multiply the average cluster size (N_c_) is the total number density of clusters, which equals to the product of the monomer density before clustering (n_0_) and the proportion of the molecules that are formed to clusters (η). The monomer density before clustering (n_0_) is proportional to the stagnation pressure (P_0_) before the gas jet, thereby we can obtain


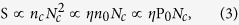


where n_0_ is the monomer density before clustering, and η is the proportion of the monomers that can form clusters. If we suppose η remains constant throughout the duration of one gas jet, then the scattering signal S is proportional to the average cluster size N_c_.

## Experimental set-up

**Figure 1:**
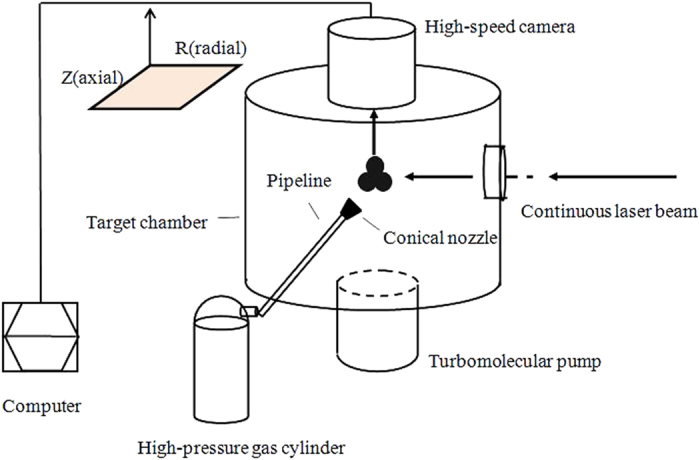
The schematic diagram of this experiment.

The experimental set-up is depicted in Fig. 1. In this study, Z represents the gas jet direction (axial), R represents the laser beam direction (radial), and the axial distance is defined as the distance between the nozzle and the center of the laser beam. And the results are discussed in RZ coordinate system. The gas jet is generated along the axial direction and the continuous laser beam perpendicular to the gas jet is focused into the vacuum chamber along the radial direction to interact with clusters and generate the Rayleigh scattering photons. The power of the laser is fixed at 10 W, the wavelength is 445 nm and the spot diameter is about 5 mm. The laser beam and the scattered light beam are shown by thick black arrows in Fig. 1. A UX50 high-speed camera from Photron Corp is used to capture the 90^o^ Rayleigh scattering lights on the top side of the vacuum chamber. The reflected lights from the wall of vacuum chamber have been disposed to minimize the background noise. The resolution of the camera is 1280*1024, the imaging speed is fixed at 1000 fps (frame per second), and the shutter speed is set to 1/1000 second to acquire entire evolution process.

The argon and methane gas with 99.999% purity are employed to produce pure clusters and minimize the influence of impurities. The stagnation pressure of the gas can be adjusted from 10 to 48 bar. A 1500 L/s turbomolecular pump is used to supply a vacuum pressure of approximately 2E-4 Pa. Moreover, the stagnation temperature is maintained at approximately 293 K in the entire test duration. The conical nozzle can be moved along the axial direction in the range of 10–180 mm. The orifice diameter and half cone angle of the nozzle is 0.5 mm and 45^o^, and the total length of the nozzle is about 3 mm. In addition, the nozzle is opened by a high-speed pulsed solenoid valve from Parker Hannifin Corp. In this study, the open time of the nozzle is fixed to 20 milliseconds (ms) and 20 Rayleigh scattering images are acquired by the high-speed camera for each gas jet.

## Results and Discussion

### Large cluster tracks

**Figure 2:**
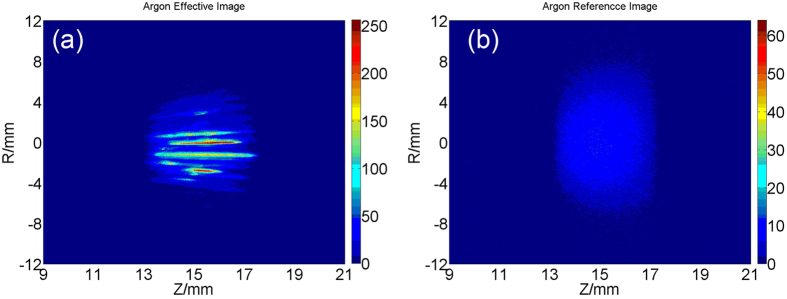
An effective image (**a**) and the corresponding reference image (**b**) of argon clusters.

For each gas jet 20 scattering lights images are acquired by the high-speed camera, normally 1~6 of the 20 images contain cluster tracks, which are used as “effective images” to analyze the properties of large cluster. And the rest images without cluster tracks are used as “reference images” to represent the average clusters. The typical “effective image” that contains cluster tracks and corresponding reference image for argon are shown in Fig. 2(a,b). The intensity of the “reference image” is proportional to the average cluster size, and the intensity of the track is proportional to the size of the clusters inside the track. Since the intensity of the track is much bigger than that in the “reference image”, we can infer the cluster size of the track is much larger than the corresponding average, which means the tracks are composed of large clusters.

**Figure 3:**
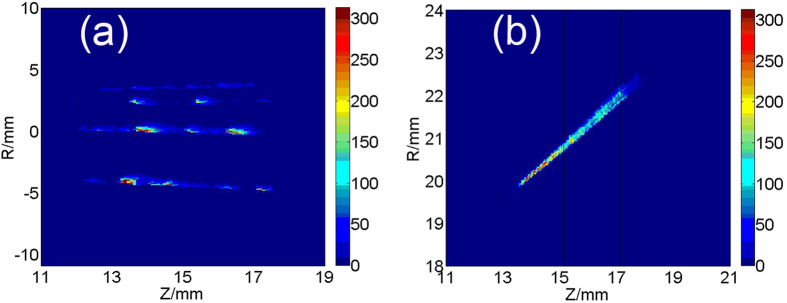
An image of methane tracks (**a**) and the combined methane track of three consecutive images (**b**).

As shown in Fig. 2(a), most argon tracks are continuous and the intensity of them changes gradually, while the majority of the methane tracks are separated into discrete fractions and the intensity of them alters periodically along the flight path as shown in Fig. 3(a). The broken and discrete process is clearly imaged for methane clusters, indicating that methane clusters may be very unstable to fragment into small pieces. It is hard to explain why the intensity alters periodically. We thought possibly it is caused by the asymmetric structure of the large methane cluster, but more studies are needed to verify.

Three consecutive tracks of the same methane clusters are acquired at axial position about 15 mm and radial position about 21 mm during one gas jet. The three images are combined together, and the combined tracks are shown in Fig. 3(b). The two boundaries of the three tracks are at the axial positions of 15 mm and 17 mm, which are shown by two black lines in Fig. 3(b). The length of the middle track is about 2.5 mm, as the shutter time for one image is 1 millisecond, then the axial velocity of the clusters can be estimated to be less than 2.5 m/s, which is far smaller than the limit supersonic velocity of methane which is 1140 m/s at room temperature (
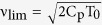
, where C_p_ is the specific heat ratio at constant pressure (J. K^−1^. Kg^−1^), T_0_ is stagnation temperature (Klevin)) before the gas jet. The opening angle of this track to the center streamline is about 54^o^, which is much bigger than the half opening angle of the nozzle which is only 45^o^.

**Figure 4:**
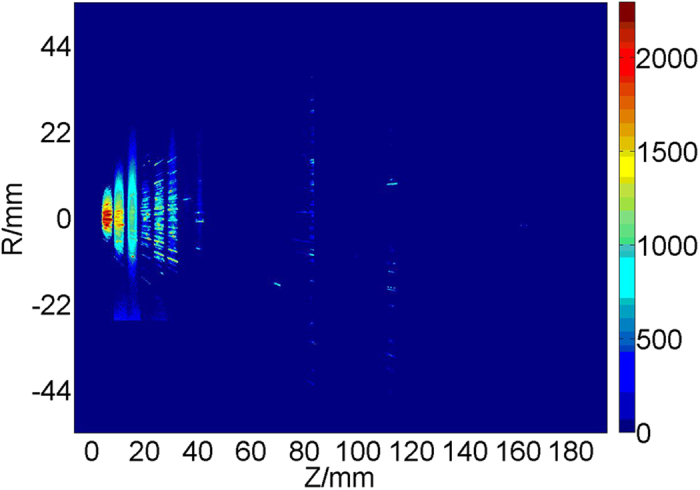
The argon cluster tracks at different axial positions.

The argon track images at different axial position are combined together into one image, which is shown in Fig. 4. It is found that the cluster tracks are distributed within the wide axial range of 10 to 180 mm and the extended line of the tracks on both sides are intersected to the same point. This verifies the results that the gas jet expends out the nozzle conically and the tracks are large clusters.

**Figure 5:**
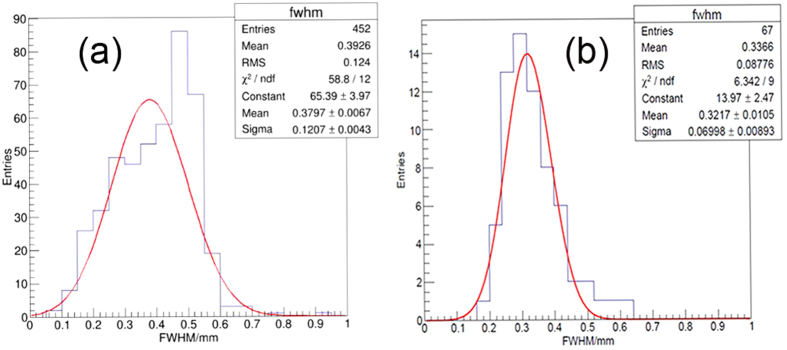
The histogram of FWHM of argon (**a**) and methane (**b**).

The full width at half maximum (FWHM) of the tracks are calculated, which is acquired by 1D summation of the pixels along the flight path of the track and get the intensity variation curve perpendicular to the flight path. The curve is fitted with the Gaussian function, and FWHM is calculated from the fitted Gaussian function. The FWHM was used to represent the width of tracks and the peak value was chosen to show intensity of tracks. However no distinct relationship between the width and intensity was found, hence the width of the track cannot be used to represent the cluster size. The FWHM distribution for argon and methane is shown in Fig. 5(a,b). The Gaussian function is used to fit the distribution, and it is found that the mean FWHM is about 0.38 ± 0.12 for argon and 0.32 ± 0.07 for methane. In this test one pixel represents 0.1 mm, which means the spatial resolution of the camera is about 0.1 mm, which is in agreement with the RMS (Root Mean Square) value of the FWHM distribution. However, we believe the width of the track gives one hint about the number of clusters inside the track, and we can imagine there are fewer clusters for the methane track than the argon track because the FWHM is smaller for methane than argon.

The cluster sizes of these large clusters can be estimated by comparing the intensity of the tracks in the “effective image” and that in the “reference image” at the same position, whereas the intensity of the “reference image” can be calibrated by the average cluster size of the gas jet.

### Average cluster size

The average cluster size is required to calculate the size of the track. One simple method to estimate the average cluster size is introduced by refs 24,25, which is used broadly by many groups^26^,^27^,^28^. When the relationship between the Rayleigh scattering signal and the stagnation pressure are tested, the average cluster size at different stagnation pressure can be easily calculated by supposing that the minimum detectable cluster size is 100 for the Rayleigh scattering method, which means the average cluster size is 100 when the Rayleigh scattering signal is just above the noise level. In ref. 22, the average cluster size is assumed to be about 100 at the “reasonable” onset point of clustering (when the signal-to-noise ratio ≈2) when the wavelength of the scattering light is 532 nm. The detectable threshold for argon cluster is supposed to be 100 when 532 nm and 526 nm laser are used in refs 25 and 26 respectively. In this work, the average cluster size is measured by using one 532 nm quasi-continuous laser with 5 kHz frequency and one PMT from Hamamatsu. And we suppose the cluster size is 100 when the signal to noise ratio is two. The PMT was set on the top of the vacuum chamber to replace the high-speed camera. The high speed camera is not suitable for this method for it is not sensitive to weak lights and normally the photomultiplier tube (PMT) is used instead.

**Figure 6:**
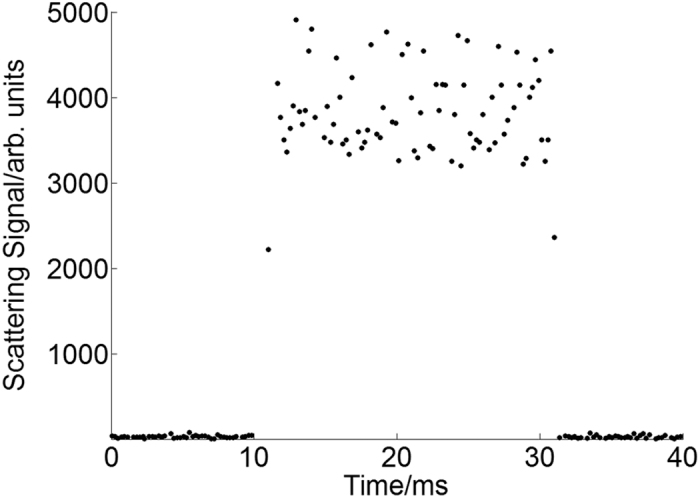
Typical Rayleigh scattering signal acquired by PMT and quasi-continuous laser.

The typical Rayleigh scattering signal at the axial position of 20 mm and stagnation pressure of 35 bar is shown in Fig. 6. The valve is opened during 10 ms and 30 ms. It is found that the Rayleigh scattering signal rises up very quickly once the valve is opened and keeps almost constant during the open period of 20 ms, this means the clusters keeps almost the same average size during the whole gas jet period, which is consistent with the result of the high speed camera.

From formula (3) we obtain S ∝ *ηPN*_*c*_, if we suppose η remains constant for different stagnation pressures, then the average cluster size is given by:


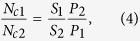


When the average cluster size Nc_1_ at stagnation pressure P_1_ is known, then the average cluster size at other stagnation pressure can be calculated easily from formula (4).

In this experiment we suppose N_c_ of argon is 100 when the scattering signal is about two times of the background noise, which happens when the stagnation pressure is about 10 bar. Since the molecular diameter of methane is larger than that of argon, the minimum detectable cluster size of methane should be smaller than argon for the same Rayleigh scattering system. The same minimum detectable cluster size of 100 is used for methane in this work for conservative purpose. And N_c_ of methane is supposed to be 100 when the stagnation pressure is about 13 bar. When the scattering signal versus stagnation pressure is tested, the average cluster size for different stagnation pressures can be calculated from formula (4).

**Figure 7:**
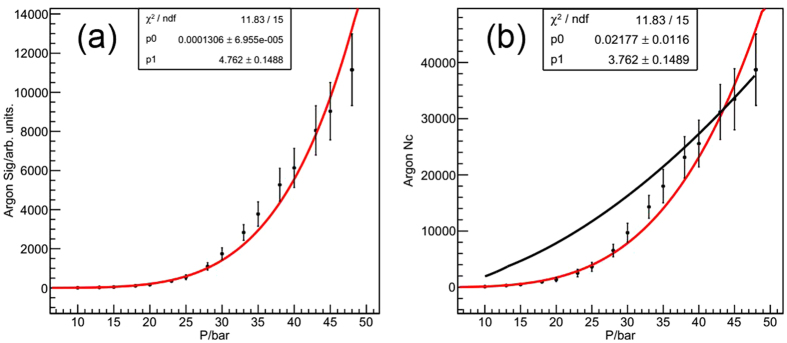
The Rayleigh scattering signal (**a**) and average cluster size (**b**) versus stagnation pressure for argon.

**Figure 8:**
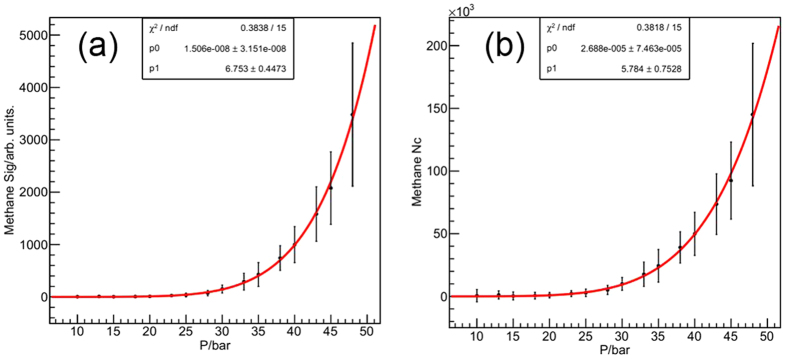
The Rayleigh scattering signal (**a**) and average cluster size (**b**) versus stagnation pressure for methane.

The Rayleigh scattering signal and the average cluster size at different stagnation pressure for argon are shown in Fig. 7(a,b), and the power function is used to fit the curve. The fitted coefficient of average cluster size versus pressure curve is a little bigger than that of Hagena. The Hagena curve is shown in black in Fig. 7(b) and it is found that the tested average argon cluster size is smaller than that calculated by the scaling law of Hagena (formula 1) at lower stagnation pressures but consistent with each other at higher pressures. This means the scaling law might overestimate the cluster size at lower stagnation pressure, and verifies that this method is effective at higher pressures. The Rayleigh scattering signal and the average cluster size versus stagnation pressure for methane are shown in Fig. 8(a,b). The fitted coefficient of N_c_ is much bigger than that of argon^29^, indicating that the average size of methane cluster will increase much heavily for higher stagnation pressure. The fitted coefficient is 5.78, which happens to be the mean value of the two coefficients of 3.8 and 7.6 in ref. 20.

The average cluster size of argon and methane at all axial positions can be calculated from the formula (3), at the stagnation pressure of 25~45 bar. The results of argon are in agreement with that in the scaling law of Hagena (1), which indicates the Rayleigh scattering system works properly and the results are reliable.

### Large cluster size distribution of the tracks

The size ratio (R_SR_) is defined as the ratio of large cluster size of the track in effective image over the average cluster size in reference image. If the average cluster sizes (N_c_) are acquired then the large cluster sizes (N_big_) can be calculated from formula (5).


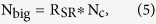


For each cluster track, the size ratio can be given by comparing the intensity of the cluster track in effective image and that at the same position in reference image, and the average cluster size can be calculated from the formula (4), therefore the large cluster size can be calculated out.

**Figure 9:**
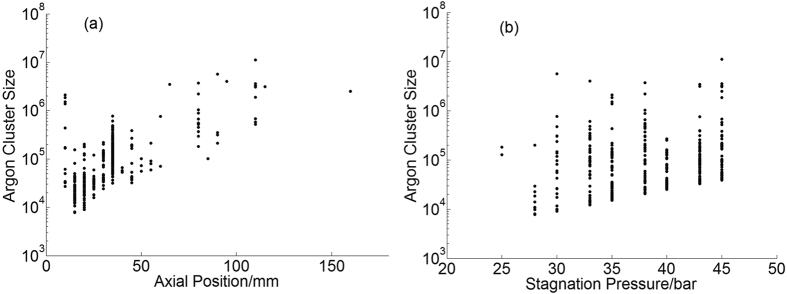
The large cluster size of argon versus the axial position (**a**) and stagnation pressure (**b**).

**Figure 10:**
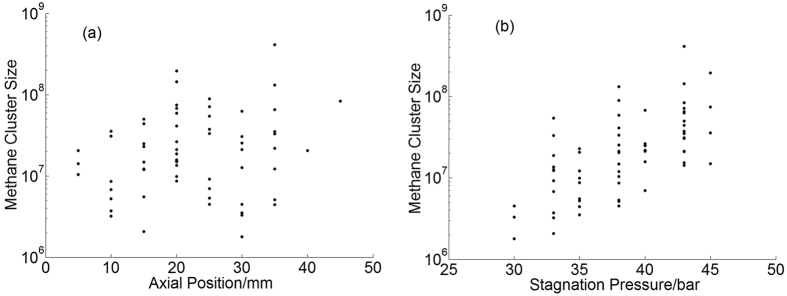
The large cluster size of methane versus the axial position (**a**) and stagnation pressure (**b**).

According to the formula (5) the large cluster sizes of argon and methane lie in the range of 7E + 03 ~ 1E + 07 and 2E + 06 ~ 4E + 08, respectively. By combining the error of the average cluster size and the size ratio, the relative error of large cluster size is calculated to be about 20% for argon and 40% for methane. The large cluster size at different axial position and stagnation pressure for argon are shown in Fig. 9(a,b), the Y axis is shown in logarithmic coordinate. There are lots of dots for each axial position in Fig. 9(a) which are corresponding to cluster tracks at different radial position. While a series of dots for each stagnation pressure in Fig. 9(b) corresponding to the tracks at different axial position. The size of large argon cluster exhibits a weak dependence both on the axial positions and stagnation pressures. This means large argon clusters can be found even at lower stagnation pressure or bigger axial positions where the gas density is lower. As for large methane clusters, the sizes are irrelevant to the axial position but have a strong growing trend with the stagnation pressure as shown in Fig. 10(a,b). But different from the argon cluster, there is hardly any methane cluster track when the axial position is bigger than 45 mm. This is possibly because the methane cluster is easier to fragment after long flight trip.

**Figure 11:**
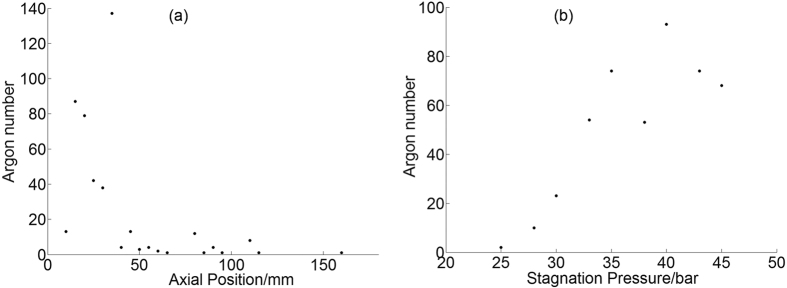
The number of argon tracks versus the axial position (**a**) and stagnation pressure (**b**).

**Figure 12:**
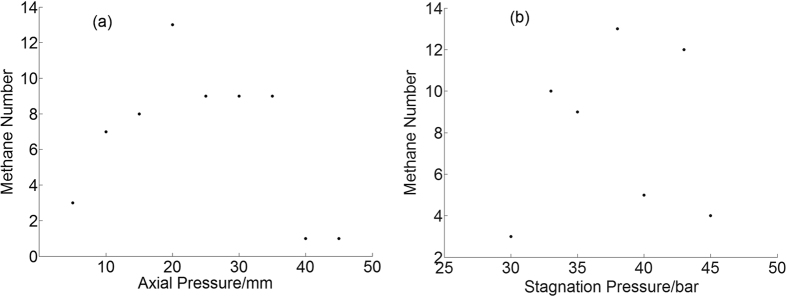
The number of methane tracks versus the axial position (**a**) and stagnation pressure (**b**).

Different from the size distribution, the number of tracks is strongly dependent on the axial position or stagnation pressure. The number of tracks versus axial position and stagnation pressure curve for argon and methane are shown in Figs 11 and 12. It is found from Fig. 11(a) that most of the large argon clusters are formed within the axial range of 15 mm to 35 mm, and the number starts to decrease sharply after that, which means these large clusters are unstable and easily to fragment into small pieces. It is found from Fig. 11(b) that more large argon clusters are formed at higher stagnation pressures when the average cluster size is higher. However no distinct relationship is found between the number of methane tracks and the stagnation pressure in Fig. 12(b). More tracks are available at the center of the gas jet both for argon and methane. The total number of methane tracks is only 1/6 of argon tracks, and it is found in Fig. 12(a) that the maximum tracks are available at axial distance of 20 mm, which decreases sharply after 40 mm and none is found after 45 mm. Different from the fact that the number of argon tracks increases linearly with the stagnation pressure, the number variation is heavier for methane tracks and no distinct trend can be found.

## Discussion

Considering the large cluster size and number of tracks, we can conclude that at higher stagnation pressure, much more large argon clusters are formed but the size only increase slightly, while much larger methane clusters are found but the number doesn’t change greatly. This means the argon cluster is inclined to increase the number, while the methane cluster tends to increase the size. The total number of argon tracks is about six times more than methane, while the cluster size of argon is about three times less than methane. Since the scattering intensity of argon is larger than methane at the same stagnation pressure, we can infer that the “number effect” is stronger than the “size effect”.

Most of the methane cluster tracks are separated into discrete fractions and the intensity of them alters along the flight path. This phenomenon indicates that these methane clusters may be very unstable to fragment into small pieces. It is hard to explain why the intensity alters periodically, and more studies are needed to verify that.

## Conclusion

In conclusion, we have demonstrated an optical method based on Rayleigh scattering for measuring the cluster tracks in gas jet. The tracks are composed of large clusters, and the sizes of large clusters for argon and methane lie in the range of 7E + 03 ~ 1E + 07 and 2E + 06 ~ 4E + 08, respectively. The sizes of large argon clusters exhibit a weak dependence both on the axial positions and stagnation pressures, whereas the sizes of large methane clusters are irrelevant to the axial position but have a strong growing trend with the stagnation pressure. The methane clusters are very unstable and easily to fragment into small pieces in comparison with argon clusters. Special methane clusters at the axial position of 15 mm and radial position of 21 mm which may fly at the axial velocity of less than 2.5 m/s are also found. The method can be setup simply and operated easily, which is very suitable for laser-cluster interaction experiments and has broad application prospects in the cluster physics and nuclear fusion fueling field.

## Additional Information

**How to cite this article**: Chen, Z. *et al*. The optical measurement of large cluster tracks in a gas jet. *Sci. Rep.* **6**, 32391; doi: 10.1038/srep32391 (2016).
